# The relationship between adult hippocampal neurogenesis and cognitive impairment in Alzheimer's disease

**DOI:** 10.1002/alz.14179

**Published:** 2024-08-21

**Authors:** Joris N. Geigenmüller, Atefe R. Tari, Ulrik Wisloff, Tara L. Walker

**Affiliations:** ^1^ Faculty of Science University of Amsterdam Amsterdam The Netherlands; ^2^ The Cardiac Exercise Research Group at Department of Circulation and Medical Imaging Faculty of Medicine and Health Sciences Norwegian University of Science and Technology (NTNU) Trondheim Norway; ^3^ Department of Neurology and Clinical Neurophysiology St. Olavs University Hospital, Trondheim University Hospital Trondheim Norway; ^4^ Clem Jones Centre for Ageing Dementia Research Queensland Brain Institute The University of Queensland Brisbane Australia

**Keywords:** adult hippocampal neurogenesis, Alzheimer's disease, cognition, hippocampus, learning and memory

## Abstract

Neurogenesis persists throughout adulthood in the hippocampus and contributes to specific cognitive functions. In Alzheimer's disease (AD), the hippocampus is affected by pathology and functional impairment early in the disease. Human AD patients have reduced adult hippocampal neurogenesis (AHN) levels compared to age‐matched healthy controls. Similarly, rodent AD models show a decrease in AHN before the onset of the classical hallmarks of AD pathology. Conversely, enhancement of AHN can protect against AD pathology and ameliorate memory deficits in both rodents and humans. Therefore, impaired AHN may be a contributing factor of AD‐associated cognitive decline, rather than an effect of it. In this review we outline the regulation and function of AHN in healthy individuals, and highlight the relationship between AHN dysfunction and cognitive impairments in AD. The existence of AHN in humans and its relevance in AD patients will also be discussed, with an outlook toward future research directions.

**Highlights:**

- Adult hippocampal neurogenesis occurs in the brains of mammals including humans.
- Adult hippocampal neurogenesis is reduced in Alzheimer's disease in humans and animal models.

## 1 INTRODUCTION

Over half a century ago it was discovered that, in contrast to the previous consensus that mammalian neurogenesis only occurs during central nervous system development, new neurons are generated throughout adulthood.^1^ The adult mammalian brain contains at least two main niches capable of producing new neurons. These include the subventricular zone adjacent to the walls of the lateral ventricles and the subgranular zone (SGZ) of the hippocampal dentate gyrus (DG).^2^ Adult‐born hippocampal neurons integrate their synapses into circuits of the mammalian hippocampus, where they contribute to structural and functional plasticity, supporting cognitive functions such as spatial learning, pattern separation, and memory, as well as mood regulation.^3^, ^4^, ^5^ In rodents, adult neurogenesis in the subventricular zone provides neurons that migrate along the rostral migratory stream to the olfactory bulb, where they contribute to processing sensory information.^6^, ^7^ Another pivotal function of both rodent adult hippocampal neurogenesis (AHN) and subventricular zone neurogenesis is the contribution of newborn neurons in response to brain injury.^8^, ^9^, ^10^, ^11^ This functional capacity is retained in neurogenic niches despite an age‐associated decline in adult neurogenesis levels.^3^ Interestingly, dysfunction of AHN is implicated in age‐associated neurodegenerative disorders such as Alzheimer's disease (AD).^12^ This disease is characterized by amyloid beta (Aβ) and Tau depositions in the brain and neurodegeneration, which are thought to underlie classical AD symptoms such as memory impairment.^13^, ^14^ The hippocampus is one of the first brain regions to be affected by AD pathology and loss of function.^13^ Crucially, AHN levels decline before the onset of hippocampal functional impairments typical of AD in certain AD mouse models (see Table 1 for an overview of the AD mouse lines and corresponding AHN alterations).^12^ Moreover, elevated levels of AHN markers are found in non‐demented people with Tau and Aβ depositions (AD pathological diagnostic criteria) in their brains.^15^ Individuals harboring these depositions normally become affected by dementia symptoms that are typical of AD.^14^ This raises the question of how dysfunction in AHN could contribute to cognitive impairments in AD, and if enhancing AHN could protect against dementia. Recent rodent studies have shown that aging or AD‐induced impairments in performance in cognitive tasks involving the hippocampus can be ameliorated by stimulating AHN.^16^, ^17^, ^18^, ^19^ Therefore, elucidation of the molecular mechanisms that regulate AHN, and its impairment in AD may provide novel therapeutic targets and strategies for ameliorating AD‐related cognitive dysfunction.

**TABLE 1:** AD models and their reported deficits in AHN.

AD model	Onset Aβ pathology	Onset AHN changes	Markers used	Source
3xTg‐AD	Hippocampal Aβ deposits at 6 months, Hippocampal intraneuronal Aβ at 2 months, extracellular Aβ plaques at 9 months Hippocampal intraneuronal Aβ at 6 months, extracellular Aβ plaques at 9 months	↓ 1 month Female ↓ 4 months, male ↓ 9 months ↓ 3 months	Sox2+ HH3+ BrdU	^20^ ^21^ ^22^
5XFAD	Intracellular Αβ accumulation in DGCs at 6 weeks, hippocampal Aβ plaques at 16 weeks Hippocampal Αβ deposits at 3 months	↓ 6 weeks ↓ 2 months ↓ 4.5 months	DCX DCX BrdU, NeuN, DCX	^23^ ^24^ ^18^
*APP/PS1*	Hippocampal Aβ deposits at 3 months	↑ 9 months	BrdU	^25^
**APP_751SL_/PS1KI**	Hippocampal Aβ deposits at 6 months	↓ 2 months	DCX	^26^
**App^NL‐G‐F^**	Hippocampal Aβ deposits at 6 months	↓ 6 months	BrdU BrdU	^27^ ^19^
APP^swe^PS1^ΔE9^	Hippocampal Aβ deposits at 4‐5 months	↓ 2 months	DCX, BrdU	^28^
APP.V717I	Hippocampal formation Aβ deposits at 10 months	↑ 10 months	DCX	^29^
*J20*	Hippocampal Aβ deposits at 5 months	↓ 7‐11 weeks ↑ 3 months, ↓ 5 months	DCX, Ki67 BrdU, PSA‐NCAM, NeuN, Ki67	^30^ ^31^
*PDAPP*	Hippocampal Aβ deposits at 12 months	↓ 12 months	BrdU	^32^
*PDGF‐APPSw,Ind*	Aβ deposits at 12 months	↑ 3 months	BrdU	^33^
Tg2576	Hippocampal and cortical Aβ deposits at 9‐12 months	Proliferation ↑ 3 months, survival ↓ 3 months	BrdU	^34^
TgCRND8	Hippocampal and cortical Aβ deposits at 3 months	↓ 3 months	BrdU, DCX	^35^

**Abbreviations:** Aβ, amyloid beta; AD, Alzheimer's disease; AHN, adult hippocampal neurogenesis; BrdU, bromodeoxyuridine; DCX, doublecortin; DGC, dentate granule cell.

## 2 RODENT AHN: FROM STEM CELL TO NEURON

**FIGURE 1:**
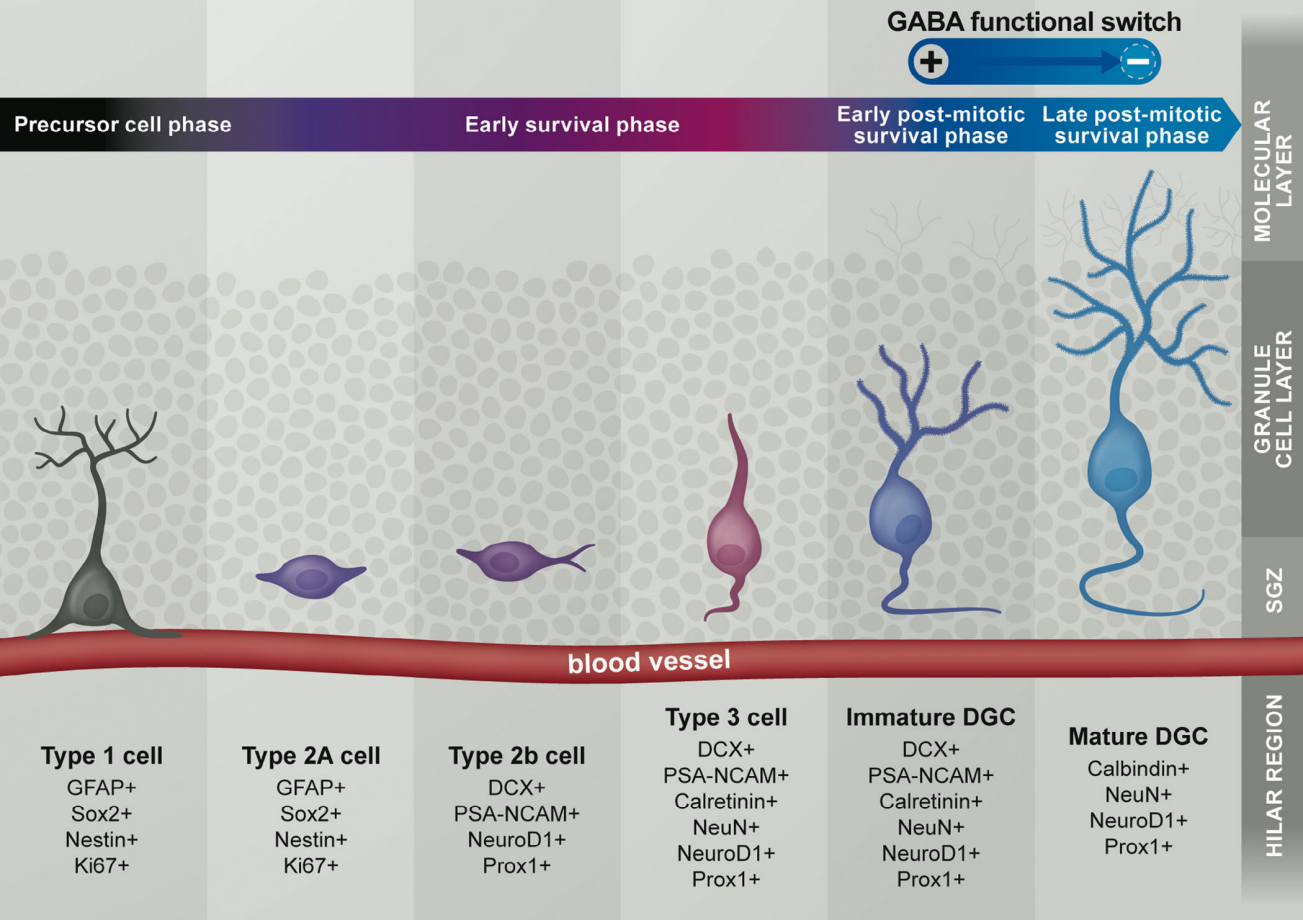
The AHN trajectory in mice from type 1 stem cells to mature dentate granule cells, along with the associated cell markers per cell type. AHN, adult hippocampal neurogenesis; SGZ, subgranular zone.

AHN is a well‐conserved mechanism across mammals and occurs in rodents according to the trajectory illustrated in Figure 1.^36^ Neural stem cells, also commonly but not exclusively referred to as type 1 cells, reside in the SGZ while their single radial apical process connects with the vasculature in the molecular layer of the DG.^37^ The neural stem cells express the astrocytic marker glial fibrillary acidic protein (GFAP), as well as the (neural) stem cell markers sex‐determining region Y‐box (Sox) 2 and Nestin, but are negative for the astrocytic marker S100β.^38^, ^39^, ^40^, ^41^ Through asymmetrical division, activated stem cells give rise to proliferating precursor cells (type 2 cells), that lose their radial process and form tangential processes.^40^, ^42^ The proliferating precursor cells are characterized by a high initial level of proliferation and subsequent differentiation and can be divided into two subcategories: type 2A and type 2B.^37^ Type 2A precursor cells retain expression of the glial marker GFAP, Nestin, Sox2, and the proliferation marker Ki67.^40^, ^43^, ^44^ These cells give rise to type 2B precursor cells which lose their GFAP expression and downregulate Nestin and Sox2.^40^ Consequently, they start expressing neuronal lineage markers such as doublecortin (DCX), prospero homeobox protein 1 (Prox1), neurogenic differentiation 1 (NeuroD1), and polysialylated neuronal cell adhesion molecule (PSA‐NCAM).^40^, ^44^ Type 2B cells generate immature neurons, also called neuroblasts or type 3 cells, which are characterized by their radial migration into the SGZ where they start to reform an apical process.^40^ The generation of immature neurons marks the final stage of the early survival phase that follows the precursor phase. During this period, cells enter their post‐mitotic state (i.e., leave the cell cycle) and begin to express the mature neuronal marker NeuN and the calcium binding protein calretinin.^37^ The early survival phase takes place from type 1 cells until type 3 cells enter their post‐mitotic state.^45^ During this phase, cells in the neurogenic trajectory are exposed to apoptotic factors, causing around 60% of them to die.^38^, ^45^ Cells that survive this wave of apoptosis are called immature dentate granule cells (DGCs) and enter the post‐mitotic maturation phase.^37^ During the early post‐mitotic maturation phase, the apical process the calretinin+ NeuN+ immature DGCs starts to elongate, and their soma radially migrates to the inner granule cell layer. They also form dendritic spines and glutamatergic synapses.^4^, ^37^ During the final phase of AHN, called the late maturation phase or late survival phase, the newborn DGCs are structurally integrated into the hippocampal circuitry.^37^ At this point, new DGCs lose their calretinin expression and become calbindin positive.^37^

## 3 FUNCTION OF AHN

Although DGCs generated during development and adulthood connect to the same circuits, AHN is considered to be functionally distinct from developmental neurogenesis.^46^, ^47^ During an initial period of functional dissimilarity to mature DGCs (referred to as the critical period), immature adult‐born DGCs possess unique electrophysiological properties (i.e., hyperexcitability and unresponsiveness to gamma‐aminobutyric acid [GABA]‐ergic inhibition).^5^, ^47^ These are thought to contribute to specific cognitive functions involving the DG.^5^, ^47^ Rodent studies employing enhancement, suppression or ablation of AHN, as well as electrophysiological recordings in humans, have elucidated several AHN‐associated functions, including spatial and temporal pattern separation, memory consolidation, and memory clearance.^48^, ^49^, ^50^ Pattern separation is a crucial cognitive function, acting to counter interference between memories with high similarity by enhancing dissimilarity in their outputs.^49^ The DG contains cells that bear engrams, that is, indexes for the activation of cells in a specific spatiotemporal pattern that together encode a memory. An engram can be activated when partial memory clues are given as input, producing a specific memory as output. The sparsity of DGCs that encode entorhinal cortex inputs enhances the specificity of memories, that is, pattern separation, allowing for memories with overlapping information to be consolidated. On a cellular level, pattern separation is thought to be enhanced by AHN‐induced inhibition of mature DGCs and Cornu Ammonis (CA)3 pyramidal neurons, which results in their elevated signaling sparsity.^5^, ^48^, ^49^, ^50^ Aside from feedforward inhibition of CA3 pyramidal neurons and feedback inhibition of mature DGCs, adult‐born immature DGCs display synaptic competition with mature DGCs.^50^ Through this modulation or elimination of existing synapses, adult‐born immature DGCs have the potential to modulate engrams that allow for pattern completion upon partial memory clues. In this way, engrams can be updated in the face of new conflicting information. Conversely, behavioral paradigms such as contextual fear conditioning show an effect of AHN modulation in memory discrimination, where ablation or enhancement of AHN impairs or improves discrimination between similar contexts in rodents, respectively.^49^ Epp et al. employed the Morris water maze paradigm to investigate the effects of exercise‐mediated AHN enhancement on pattern completion and memory clearance in mice.^51^ During the first platform localization task, the exercise group performed worse than the control (sedentary) group, indicative of increased memory clearance in the exercise group.^51^ Strikingly, during the platform reversal learning task, the exercise group showed an improvement in task performance (i.e., pattern separation) compared to the sedentary group, highlighting that elevated AHN decreases pattern completion performance but improves pattern separation performance. Corroborating these findings, Nakashiba et al. showed that adult mice in which old but not young DGCs are inhibited present deficits in pattern completion in the Morris water maze. Although pattern separation was not negatively affected in this paradigm, additional removal of young DGCs in these mice did impair pattern separation.^52^ Adult‐born immature DGCs are also suggested to encode the temporal context of memories due to their hyperexcitable properties during the aforementioned critical period.^5^, ^53^ This theory posits that adult‐born immature DGCs encode temporal associations between separate memory events that were integrated during their critical period between 2 and 6 weeks of age.^5^, ^53^ The signal output from the DG of recently formed memories would thereby be more similar than that of older memories. Thus, continuous AHN supports temporal memory separation: the period of hyper‐excitability of adult‐born DGCs acts as a context‐specific time stamp in engrams.

In summation, AHN maintains the balance between immature DGC‐mediated pattern separation, mature DGC‐mediated pattern completion, and memory clearance.^48^, ^52^ This supports cognitive functions such as pattern separation, spatial navigation, and contextual memory encoding.^5^, ^54^

## 4 HUMAN ADULT NEUROGENESIS

Although the existence of adult neurogenesis in rodents has been widely accepted, reports of continued human adult neurogenesis remain a topic of debate. No consensus exist regarding the existence of adult ventral subventricular zone neurogenesis in humans, although it is generally accepted that the adult human olfactory bulb does not receive new neurons from the subventricular zone.^11^ In contrast, AHN is proposed to contribute to certain hippocampal cognitive functions such as contextual and spatial pattern separation throughout the human lifespan, and to play a role in AD pathology.^12^, ^49^ In humans, the existence of AHN has been reported using techniques such as 5‐bromo‐2′‐deoxyuridine (BrdU) staining and retrospective birth dating using radiocarbon ^14^C.^12^ However, technique‐specific limitations have contributed to a lack of uniform results. For example, lipofuscin, which is abundant in the brains of older individuals, causes background autofluorescence which could hinder BrdU detection.^55^ Radiocarbon dating in turn can be prone to background carbon contamination, potentially contributing to inconclusive findings in studies employing this technique.^56^ Furthermore, BrdU staining used to assess proliferation may generate a positive signal upon DNA repair and methylation, possibly obscuring an AHN‐induced positive signal.^57^ In general, research on human AHN is hindered by a lack of non‐invasive techniques to assess the genesis and migration of newborn neurons. In vivo and ex vivo techniques employed in animal studies such as genetic ablation of AHN and the culturing of hippocampal precursor cells are not suitable for research in humans. Moreover, the resolution of neuroimaging techniques such as magnetic resonance imaging is currently too low to identify DGCs formed during AHN. Therefore, research into human AHN is primarily limited to the use of *post mortem* brain tissue and neural progenitor cultures derived from patient brain tissue or induced pluripotent stem cells. As reviewed by Gault and Szele, studies of human AHN using *post mortem* brain tissue often vary in *post mortem* interval and age of the brains used, both of which are likely to cause discrepancies in results.^58^ Critically, Moreno‐Jiménez et al. have shown that the fixation procedure (e.g., fixation compound, fixation length, and storage method) are of critical importance for successful immunohistochemical staining with the markers for immature neurons, PSA‐NCAM and DCX^59^ with conflicting results being observed when using these markers in human tissue.^60^, ^61^ Whereas Boldrini et al. reported stable expression of DCX+ PSA‐NCAM+ cells in the DG of humans aged 14‐79, a publication by Sorrells et al. the same year failed to find these cells in DG of humans aged 18–77.^60^, ^61^ It should be noted that methodological differences between these studies may contribute to this discrepancy. Unlike Boldrini et al., Sorrells et al. did not perform antigen retrieval for DCX and PSA‐NCAM.^60^, ^61^ Moreover, Sorrels et al., used samples that were fixed for more than 48 hours.^61^ The Llorens‐Martin group, however, report that short fixation times are necessary, not only to detect DCX and PSA‐NCAM, but also a wide variety of other AHN markers.^62^, ^63^ In addition, the brain tissue used Sorrells et al. had a *post mortem* interval of less than 48 hours, whereas Boldrini et al. used tissue with a *post mortem* interval of 4–26 hours.^60^, ^61^ These factors may very well have contributed to the reported lack of DCX+ PSA‐NCAM+ cells in the DG by Sorrells et al.^61^ Other contemporary in vitro techniques used to investigate human AHN include single‐nucleus or single‐cell transcriptomic analyses of cultured human neural progenitor cells, although studies employing these techniques have produced conflicting results.^64^ These approaches can, however, provide valuable insights into the expression profile of human adult born hippocampal neurons, including age‐related changes in DGC gene expression, allowing a more detailed definition of immature DGCs than relying solely on the expression of DCX and PSA‐NCAM. Nonetheless, it should be noted that an important limitation to these in vitro studies is the inability to accurately replicate the microenvironment of the (human) SGZ that is critical for type 1 cell maintenance, proliferation, and differentiation in vivo. This hinders proper recapitulation of factors that regulate in vivo AHN, such as active synaptic sites and vasculature.^65^, ^66^ Moreover, Tosoni et al. have emphasized that the reported transcriptional profile of (im)mature DGCs is subject to differences in the isolation method and samples.^64^ These include the *post mortem* interval, sequencing depth, the choice between single‐nucleus or single‐cell sequencing, age, and number of brains and cells and the diagnostic criteria for healthy and diseased brains used for comparisons.^64^ This most likely contributes to discrepancies in reports regarding the existence of AHN in aged humans. Nevertheless, recent studies based on immunohistochemistry and single‐nucleus transcriptomic analysis of *post mortem* brain tissue have provided robust evidence for AHN in human adults and its impairment in AD patients.^62^, ^67^, ^68^

## 5 AD

AD is a neurodegenerative disorder characterized by the progressive accumulation and spread of protein aggregates throughout the brain, coupled with neuroinflammation and neurodegeneration.^13^ These pathological changes contribute to progressive cognitive impairment such as learning and memory deficits that are typically observed in AD.^14^ The vast majority of AD cases have no clear singular (genetic) cause and are referred to as sporadic AD.^14^ However, a small subset of AD cases can be linked to a specific genetic defect (familial AD).^14^ A diagnostic criterion for AD is the presence of extracellular insoluble Aβ plaques and intraneuronal neurofibrillary tangles, which form upon aggregation of monomeric Aβ_40_ or Aβ_42_ and monomeric Tau, respectively.^69^ The spread of these aggregates occurs in an AD‐specific pattern, most commonly classified using Thal staging for Aβ (Thal stage I—V) and Braak staging for neurofibrillary tangles (Braak stage I‐VI).^69^ Notably, the spread of soluble Aβ through the brain correlates more closely with clinical symptoms of AD than the spread of Aβ plaques.^70^ The hippocampus is affected early in AD: neurofibrillary tangles first accumulate in the entorhinal cortex and spread to CA1, followed by the subiculum, remaining CA regions, and the DG.^69^, ^71^ Atrophy in the entorhinal cortex and hippocampus together with synaptic density decrease are early hallmarks of AD, correlating with a decline in cognitive function.^13^, ^72^ Human AD in vivo magnetic resonance imaging studies have reported that the loss of functional connectivity occurs in all hippocampal subregions, whereas hippocampal neurodegeneration is mostly found in CA1.^73^, ^74^ Protein depositions other than Aβ and Tau have also recently been discovered to be associated with AD, such as TAR DNA binding protein 43 (TDP‐43).^75^, ^76^, ^77^ Depositions of this protein in the hippocampus of AD patients are associated with higher rates of hippocampal atrophy in brains with moderate to advanced neurofibrillary tangle pathology, but not in brains with little to no neurofibrillary tangle pathology.^78^ The pathological features driven by AD‐associated protein aggregation are likely multifaceted and might not be uniform across AD patients and animal models, as will be discussed in detail in the following sections. Correspondingly, the presence of AD‐typical Aβ and Tau depositions has been reported in certain cognitively healthy aged individuals.^15^ Moreover, AD‐related gray matter atrophy is not uniform across AD patients and does not affect the hippocampus or cortex in all cases.^79^

## 6 AHN IS IMPAIRED IN AD RODENT MODELS

**FIGURE 2:**
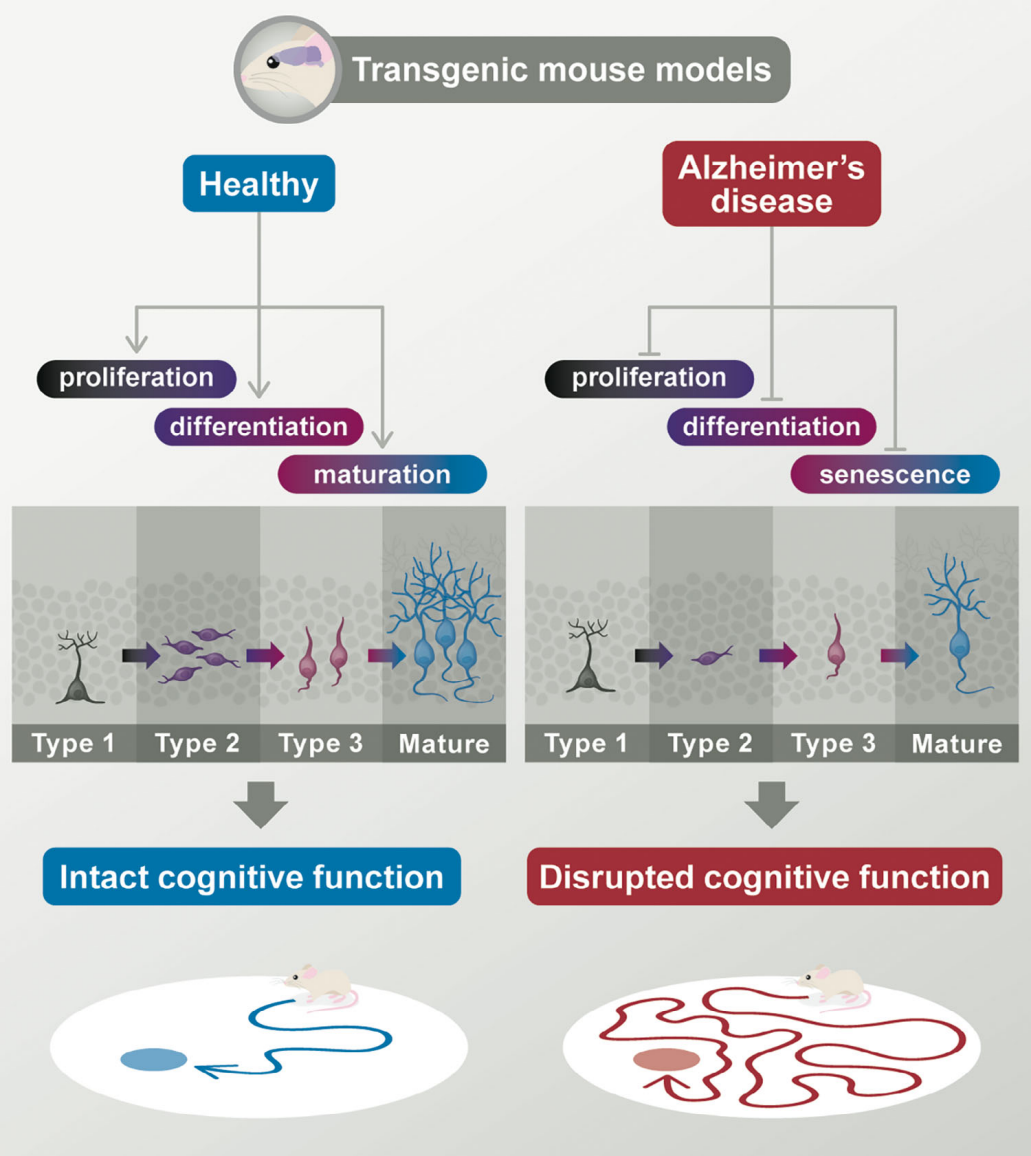
AHN and associated cognitive function is disrupted in transgenic rodent models of AD. These include changes in proliferation, differentiation, neuronal maturation, and functional changes in AHN‐associated cognitive functions such as spatial learning and memory assessed using the Morris water maze test. AD, Alzheimer's disease; AHN, adult hippocampal neurogenesis.

Crucially, impairments in AHN have been found to precede characteristic AD protein depositions or hippocampal volume decrease in various AD animal models such as 3XTg‐AD, APP/PS1 variants, 5XFAD, and others.^80^ See Table 1 for examples of some of the more widely studied transgenic AD models and associated AHN changes. These include AHN changes in proliferation, differentiation, DGC maturation, and functional changes in AHN‐supported cognitive functions as assessed by behavioral paradigms such as contextual fear conditioning and the Morris water maze^81^ (Figure 2). Crucially, besides differences in the onset of plaque pathology and changes in AHN, some models show an increase rather than a decrease in hippocampal precursor cell proliferation or differentiation.

Using the 3XTg‐AD mouse model which harbors amyloid precursor protein (APP) and Tau mutations and presents Aβ deposits and Tau phosphorylation, Liu et al. investigated impairments in AHN. The authors reported continued impaired hippocampal proliferation as determined by levels of hippocampal Sox2 and DCX as early as postnatal day 5 and 1 month, respectively.^20^ This precedes the hippocampal accumulation of intraneuronal Aβ deposits, Aβ plaques and phosphorylated Tau in this model, which are reported to begin at 6, 9 and 4 months, respectively.^22^, ^82^ Moreover, the AHN impairments occurred before the onset of hippocampal volume loss at 3 months. These findings were corroborated by Wang et al., who found that proliferation in the DG was impaired 3 months before the onset of Aβ pathology in this model.^22^ Strikingly, in the J20 AD mouse model, which expresses three APP isoforms with two human mutations, López‐Toledano and Shelanski reported an increase in BrdU incorporation, and Ki67, NeuN, and PSA‐NCAM staining in the SGZ at 3 months.^31^ This increase in markers of proliferation preceded the formation of hippocampal Aβ oligomers and plaques, which begin at 2 and 7 months, respectively. From 5 months onward, however, the J20 mice showed a decrease in hippocampal BrdU incorporation and Ki67 staining, indicating that the initial increase in proliferation was lost over time and was followed by AHN impairment. Potentially, an AHN rescue mechanism could be involved early and be lost as AD pathology increases. In the 5XFAD mouse model, Kim et al. found intraneuronal Aβ accumulation in the SGZ DGCs of 6 week old mice, 10 weeks before the formation of extracellular Aβ plaques.^23^ This was coupled with a decrease in hippocampal DCX+ cells without changes in proliferating cell nuclear antigen+ (PCNA) and Sox2+ cells. DCX is a differentiation marker, whereas PCNA and Sox2 are markers for proliferation and stem cells, respectively. This led the authors to conclude that, in young 5XFAD mice, the Aβ accumulation in SGZ DGCs impairs AHN differentiation but not proliferation. The 5XFAD model (with Cre/Lox‐based tamoxifen‐inducible neurogenesis enhancement) was used by Mishra et al. to investigate putative impairments in engram formation and activation as a result of AD pathology.^18^ First, the authors reported that the AD mice had fewer immature DGCs than their wild‐type counterparts. Using viral engram labeling combined with NeuN immunostaining, it was found that newborn DGCs were incorporated in engrams in both wild‐type and AD mice. However, the latter group showed aberrations in dendritic spine density of immature DGCs as well as reduced activation of engram circuit neurons during memory retrieval, indicative of perturbed engram formation and function. Correspondingly, this group showed cognitive deficits in memory tests compared to the wild‐type group. Tamoxifen‐based augmentation of AHN in the AD group ameliorated the perturbed engram formation and improved their engram specificity, thereby enhancing performance in AHN‐associated spatial pattern separation paradigms. Using an AD mouse model carrying a familial AD mutation in the presenilin‐1 gene, Wang et al. showed that this mutation impairs AHN‐supported contextual pattern separation. In a contextual fear conditioning paradigm, 3 month old transgenic mice showed performance deficits, coupled with a decrease in BrdU incorporation in the DG, indicative of impaired AHN.^83^ Taken together, these studies provide evidence that in various animal models, AD pathology is coupled with perturbed type 1 cell proliferation, differentiation, or maturation. This suggests that AHN impairment may contribute to AD‐related pathological hallmarks, rather than being a downstream effect of them.

## 7 AHN IS DISRUPTED IN HUMANS WITH AD

In accordance with the findings of the AD model studies described above, AD‐driven impairment of AHN has been reported in humans. In recent years, four studies analyzing the *post mortem* brain tissue of healthy aged humans and AD patients have provided robust evidence for AHN impairment in AD.^62^, ^67^, ^68^ Using *post mortem* hippocampal tissue, Moreno‐Jiménez et al. reported that the number of DCX+ neurons in the DG decreased in AD patients compared to neurologically healthy control subjects.^62^ Braak staging was used to differentiate between AD patients (Braak stages II‐VI) and healthy controls (Braak stage 0). Strikingly, the percentage of DCX+ neurons in the DG that were positive for calretinin did not differ between groups. In contrast, the percentage of DCX+ cells that were calbindin+ was lower in AD patients from Braak stage IV onward compared to the control group. This indicates that the maturation or survival of DGCs in the AHN trajectory is impaired in AD. Tobin et al. investigated the levels of AHN in the post‐mortem hippocampal tissue of healthy aged subjects and people diagnosed with AD or mild cognitive impairment, classifying patients based on cognitive status and clinical diagnosis instead of the Braak staging employed by Moreno‐Jiminez et al.^67^ The authors found a positive correlation between cognitive scores and the number of DCX+ PCNA+ cells. Although not significant, a similar trend was seen for the number of Nestin+ Sox2+ Ki67+ cells and cognitive scores. Zhou et al. used single‐nucleus RNA sequencing to identify immature DGCs in the *post mortem* hippocampal tissue of eight AD patients (seven patients with Braak stages III‐VI, one patient with unspecified Braak stage) and age‐matched healthy controls.^68^ They reported that immature DGCs accounted for 3.6% of the DGCs in healthy controls, compared to 1.1% in AD patients. Cao et al. investigated impairments in AHN using post‐mortem hippocampal tissue of AD patients, as classified by the ABC sco*ri*ng protocol that assesses Aβ deposits, neurofibrillary tangles, and neuritic plaques to obtain a score ranging from low to high.^84^ AD patients were defined as those with an ABC score of intermediate or higher, whereby healthy controls were defined as those with an ABC score of none or low. The authors found that AHN is impaired in AD, as the number of calretinin+ Prox1+ and calretinin+ DCX+ DGCs decreased in the AD hippocampus, compared to healthy controls. Interestingly, they also observed an increase in Nestin+ cells in the AD hippocampus, which they suggested was the result of elevated astrogliogenesis. The reported AD‐induced impairment of AHN in these publications was corroborated by studies investigating the performance of AD patients in cognitive tasks that are supported by AHN.^85^, ^86^, ^87^, ^88^, ^89^ This supports the evidence for AD‐induced AHN impairment in AD patients provided by immunohistochemical studies.^62^, ^67^, ^68^

The unique electrophysiological properties of immature DGCs are thought to generate a bias toward their activity over that of mature DGCs, but might also make maturing DGCs particularly vulnerable to an AD‐induced imbalance in Ca^2+^ homeostasis and excitotoxicity.^37^, ^54^, ^90^, ^91^ Moreover, changes in immature adult‐born DGCs, either due to the absence of these cells or alteration of their electrophysiological properties, likely hinder the AHN‐mediated enhancement of cognitive functions such as pattern separation. Post‐mitotic neurons re‐entering the cell cycle (i.e., de‐maturing) are proposed to adopt a senescence‐like phenotype and exacerbate AD pathology.^92^ Therefore, investigating senescence markers in the DG neurogenic niche of post‐mortem brains of AD patients in more detail could elucidate the potential involvement of immature DGCs. Examining the expression of potassium and chloride ion co‐transporters NKCC1 and KCC2, which are expressed by type 2 cells and onward and during the gamma‐aminobutyric acid (GABA) functional switch that occurs at the DGC maturation phase^4^, ^44^, ^93^ respectively, in putative (pseudo)immature DGCs could also offer more insight into the functional state of DG cells expressing immature DGC markers.

## 8 PROPOSED MECHANISMS UNDERPINNING THE AD‐DRIVEN IMPAIRMENT OF AHN

**FIGURE 3:**
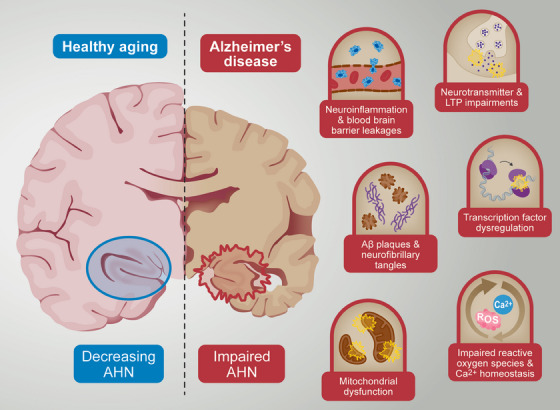
Proposed mechanisms underpinning the AD‐driven impairment of AHN. Many different regulators of AHN are proposed to be affected by AD pathology, such as mitochondrial dysfunction, impaired calcium homeostasis and reactive oxygen species, transcription factor dysregulation, neurotransmitters, and neuronal signaling impairments and Aβ plaques and neurofibrillary tangles. Aβ, amyloid beta; AD, Alzheimer's disease; AHN, adult hippocampal neurogenesis.

Many different micro‐ and macroenvironmental regulators of AHN are proposed to be affected by AD pathology, such as mitochondrial dysfunction, calcium homeostasis, transcription factors, reactive oxygen species, neurotransmitters, and neuronal signaling (Figure 3). Neuroinflammation, neurovascular changes, and loss of blood‐brain barrier integrity are also AD‐associated brain alterations that have been proposed to affect AHN. The following sections will highlight some of the potential mechanisms underpinning AD‐driven impairment of AHN.

### 8.1 Mitochondrial dysfunction and calcium homeostasis

Mitochondrial dysfunction, and elevated mitochondrial protein folding stress due to aging have been proposed to contribute to age‐related AHN decline.^94^, ^95^ Pathological Aβ disrupts membrane integrity and ion channel activity, dysregulating Ca^2+^ influx and causing elevated levels of cytosolic Ca^2+^ in neurons, thereby impairing brain derived neurotrophic factor transport and enhancing neuronal vulnerability to excitotoxicity.^70^, ^96^ Localization of Aβ in mitochondria is another hallmark of AD, causing their dysfunction and contributing to impairments in cellular reactive oxygen species and Ca^2+^ homeostasis.^97^ When Kim et al. treated human neural progenitor cells with mitoAβ^+,^ an Aβ construct that localizes in mitochondria,^23^ they found an intracellular decrease in the neurotrophin brain derived neurotrophic factor, which induces calbindin expression.^98^ Correspondingly, the mitoAβ^+^ cells in the SGZ of 5XFAD mice were positive for the stem cell markers Sox2 and GFAP but not calbindin, indicative of their impaired differentiation and maturation.^23^ Based on these results, the authors proposed that Aβ‐induced mitochondrial impairment drives the suppressed differentiation of neural progenitor cells.

### 8.2 Genetic risk factors

The human apolipoprotein isoform E (ApoE) 4 is a known genetic risk factor for AD, and the ApoE protein is also crucial for AHN.^99^ Using genetic knock‐in and knock‐out models, Li et al. showed that ApoE knock‐out or human ApoE4 knock‐in adult mice produced significant decreases in AHN compared to wild‐type controls and human ApoE3 knock‐in mice, respectively.^100^ The authors found that ApoE4 knock‐in caused a 60% increase in SGZ proliferation (measured by SGZ Ki67+ cell count) compared to wild‐type controls, ApoE knock‐out mice, and human ApoE3 knock‐in mice. Zheng et al. found that in adult 3xTg‐AD mice, ApoE4 knock‐in also causes ∼ 70% of the parvalbumin+ DG interneurons to accumulate aggregation‐prone phosphorylated Tau, resulting in a reduction in this cell population and suppression of their GABA release.^101^ GABA spillover from parvalbumin+ DG interneurons provides a tonic GABAergic signal to type 1 cells that promotes their quiescence.^102^ Moreover, GABA signal also provides an excitatory stimulus to immature adult‐born DGCs that is essential for AHN in rodents.^41^, ^103^ Thus, it follows that, in rodents, decreased levels of GABA in the DG as induced by AD pathology are coupled with an increase in type 1 cell proliferation, as well as impaired DGC maturation.

### 8.3 Disruptions in neuronal plasticity

Knock‐in of the ApoE4 mutation causes a decrease in dendritic spine density and complexity of adult‐born DGCs in the DG of mice.^104^ In the 5XFAD mouse model, Mishra et al. found that DCX+ NeuN+ immature DGCs showed a decreased dendritic spine density and transcriptional profile changes, coupled with a performance deficit in a contextual fear conditioning task.^18^ In the 3xTg‐AD model, Valero et al. reported a reduction in DCX+ cells with puncta positive for vesicular glutamate transporter and post‐synaptic density marker 95, markers for glutamatergic neurons and synaptic receptor function, respectively, in the molecular layer compared to wild‐type mice.^105^ Therefore, the reduction of DCX+ cell puncta in the molecular layer positive for these markers in the 3xTg‐AD mice is indicative of their impaired DGC maturation. Another way in which AHN is affected by AD pathology is through alterations in the long‐term potentiation of maturing adult‐born DGCs. The (Ca^2+^ influx‐mediated) autophosphorylation of calcium‐calmodulin‐dependent kinase II that causes α‐amino‐3‐hydroxy‐5‐methyl‐4‐isoxazolepropionic acid receptors to translocate to the cell membrane is impaired by AD pathology.^106^, ^107^ This leads to a decrease in presence of this receptor on the membranes at synaptic sites, impairing long‐term potentiation.^107^

### 8.4 Dysregulation of transcription factors

Transcription factors play a pivotal role in the regulation of AHN with AD‐induced alteration in their activity proposed to contribute to its dysregulation. The expression of Wingless‐related integration site (Wnt), a pivotal transcription factor in AHN regulation, decreases with age in mammals.^108^ Furthermore, the expression of the Wnt antagonists Dickkopf‐1 and secreted frizzled‐related protein 3 increases in the aging mouse brain.^109^ The effects of aging‐related decrease in Wnt signaling are exacerbated by AD pathology in various ways. The brains of AD patients show elevated levels of Dickkopf‐1 and ex vivo administration of Aβ oligomers to adult mouse hippocampal slices has been reported to cause synaptic loss through elevation of Dickkopf‐1 levels.^110^ In vitro, aged neural progenitor cells respond to stimulation with exogenous Wnt1, 3, and 3a ligands by expressing pro‐survival factor Survivin via canonical Wnt pathway activation.^111^

Another AHN‐regulating transcription factor affected by AD is bone morphogenetic protein (BMP), which is produced by DGCs to regulate type 1 cell quiescence.^112^, ^113^ Crews et al. assessed the expression of BMP6 in the hippocampus of AD patients and APP transgenic mice.^112^ In both cases they found elevated levels of BMP6 expression in the hippocampus, and that this was associated with a decrease in markers of AHN in their mouse model. In addition, Cao et al. reported increased BMP6 expression in the hippocampus of AD patients coupled with a decrease in AHN and an increase in astrogliosis.^84^

### 8.5 AD‐induced reactive oxygen species alterations and neuroinflammation

Hippocampal oxidative stress, that is, a state wherein reactive oxygen species cause cellular damage, is a hallmark of AD.^114^ In a study using *post mortem* tissue of AD patients, Cruz‐Sánchez et al. showed that the AD hippocampus is characterized by elevated markers of oxidative stress.^115^ Aβ has been reported to cause irreversible mitochondrial damage and paired mitochondrial reactive oxygen species alterations in neural stem cells.^116^ Therefore, AD pathology in hippocampal precursor cells likely influences their ability to change their functional state. Interestingly, treatment of aged but not young rat hippocampal neurons with Aβ oligomers in vitro induces an increase in reactive oxygen species generation and cytosolic Ca^2+^, highlighting the effects of age on mechanisms regulating AHN.^117^

Another manner by which exercise promotes AHN is through the reduction of neuroinflammation, which is typically seen in aging and AD.^118^, ^119^ Investigating the effects of neuroinflammation on AHN, Valero et al. used the 3xTg‐AD model as well as lipopolysaccharide‐induced neuroinflammation.^105^ They reported that the injection of lipopolysaccharide caused or exacerbated neuroinflammation in wild‐type mice and 3xTg‐AD mice. Furthermore, the lipopolysaccharide treatment caused a decrease in NeuN expression in the DG of wild‐type but not 3xTg‐AD mice. Interestingly, the administration of lipopolysaccharide in 3xTg‐AD mice caused an increase in BrdU+ NeuN+ cells in the DG, indicative of increased AHN. The neuroinflammation‐induced perturbation of neural stem cells and astrogliogenesis bias over neurogenesis is also seen in conditions of phosphorylated Tau‐induced parvalbumin interneuron functional impairment.^101^, ^120^ Moreover, neuroinflammation can cause microglia to switch from an anti‐inflammatory and anti‐oxidative state to a proinflammatory state, which promotes astrogliogenesis over neurogenesis.^121^ Zheng et al. reported that in vivo overexpression of human Tau in GABAergic interneurons in the DG of adult mice promotes astrogliogenesis at the cost of AHN.^101^ Accordingly, a decrease in AHN induced by targeting DG parvalbumin+ interneurons causes performance deficits in learning and memory paradigms.^101^, ^122^ The imbalance between neurogenesis and gliogenesis in AD has also been suggested to be affected by Aβ, with Aβ_40_ reportedly promoting neurogenesis, whereas Aβ_42_, the prevalent form of Aβ in AD, promotes gliogenesis.^123^

### 8.6 Systemic blood factors and gut microbiota

The DG may be particularly vulnerable to AD‐related blood‐brain barrier leakages and pathogenic factors in the plasma due to its extensive vascularization.^48^, ^124^ Moreover, the reduced laminin expression and altered basement membrane at the contact site between the apical processes of hippocampal neural stem cells and the vasculature allows an enhanced access of these cells to systemic factors.^125^ Using heterochronic parabiosis, Villeda and colleagues showed that blood‐borne factors mediate levels of AHN.^126^ Building on this, Zhao et al. showed that repeated administration of the plasma from young wild‐type mice to 3xTg‐AD mice can ameliorate deficits in cognitive functions supported by AHN, although they did not observe changes in AHN marker expression.^127^ To further investigate the effects of exercise‐induced changes in systemic factors, Norevik et al., attempted to transfer the neurogenic effect of exercise via the systemic environment. Plasma from healthy, young Wistar rats injected (repeated administration) into a transgenic AD rat model (McGill‐R‐Thy1‐APP) showed an increase in AHN in the AD rat brain.^128^ Similarly, De Miguel et al., observed an increase in AHN and an enhancement in memory function, in APP‐mice receiving plasma from runner mice.^129^ Similar effects were also observed by Kim et al. who found that plasma from exercised mice could ameliorate the AHN and cognitive deficits observed in 3xTg‐AD transgenic mice.^130^

The Thuret lab have recently developed a human progenitor cell assay to model the effect of systemic factors on AHN.^131^ Using this model, they showed that they could differentiate between individuals with mild cognitive impairment who either progressed to AD or remained relatively stable, thus providing a platform for early prognosis and monitoring of AD progression. Notably, they found that serum collected from individuals who subsequently deteriorated and developed AD caused the cultures to increase in cell death, decrease in cell growth, but increase in conversion from immature cells to neurons. They hypothesized that this increase in AHN may be an early compensating mechanism in AD progression.

Excitingly, recent research has shown that gut microbiota may affect AHN in AD. Grabrucker et al., showed that AD patient‐isolated gut microbiota decreased AHN and related cognition in healthy wild‐type rats.^132^ Similar effects were also observed when the gut microbiota of 5xFAD mice were transplanted to wild‐type mice, which resulted in performance deficits in AHN‐supported cognitive function, as well as a decrease in AHN.^133^ Together, these studies emphasize the important role that systemic factors play in mediating AD‐induced AHN impairments.

## 9 CONCLUSIONS AND FUTURE DIRECTIONS

Despite compelling data from animal models, only correlative evidence has been provided linking impairments in AHN in human AD patients with cognitive dysfunction. Several roadblocks must be first overcome before a more thorough understanding of the role of AHN in human AD can be reached. Non‐invasive methods to reliably assess AHN activity and changes in vivo are currently lacking, hindering the ability to establish causative links between AHN, AD pathology, and therapeutical interventions in humans. As a result, contemporary studies rely on the use of post‐mortem tissue or functional imaging to establish correlations between AD, AHN, and performance in cognitive tasks involving AHN in human adults. Although it is difficult to conclusively establish causative links using these techniques, studies with human *post mortem* brains and animal models have resulted in robust findings supportive of the persistence of AHN throughout the human lifespan and its impairment in AD. It should be noted that the current lack of reliable markers of human AHN could also impact the validity of data that bases cell type annotation of mouse‐inferred markers, as exemplified in the recent analysis by Tosoni et al.^64^ In their analysis of datasets from sequencing studies of AHN in various animals, they showed that overlap between markers of AHN is limited between humans and rodents.^64^

Although a clear AHN‐AD signature is yet to be characterized, novel proxies of how AHN is affected by the systemic environment during AD development are emerging and may serve as early biomarkers of the disease pathophysiology more than 3 years before clinical diagnosis.^131^ This trial by Maruszak and colleagues also underpins the hypothesis that an increase in AHN might be an early compensating mechanism, which is exciting to consider in future trials, particularly considering that longitudinal studies have indicated that AD‐related pathophysiological changes occur up to several decades before symptomatic onset.^131^

Further refinement of biomarkers of AHN in combination with long‐term clinical studies in well‐characterized cohorts of older adults in which some stay healthy, and some develop mild cognitive impairment and AD is required. This, in combination with blood samples collected across multiple time points (from a healthy state), and where *post mortem* brain tissue is available for validation of clinical diagnosis. The randomized exercise trial, the Generation 100 Study, in which more than 1500 older adults aged 70–77 years have been followed for 10 years, with available blood samples from baseline, 1, 3, 5, and 10 years is an ideal cohort to study disease development and progression longitudinally for up to a decade before diagnosis of AD.^134^ Furthermore, ongoing clinical trials, such as the randomized double‐blinded ExPlas Study,^135^ are currently evaluating the effects of treating AD and mild cognitive impairment patients with plasma transfusion from young, healthy, fit, and exercising adults.^135^, ^136^, ^137^ With exercise training, and high age‐relative cardiorespiratory fitness levels emerging as the most promising preventive measures against AD, as well as being a potent mediator of AHN, ExPlas, and the Generation 100 Study may open the possibility to study the links between AHN, AD pathology and therapeutical interventions in humans.^138^, ^139^, ^140^

Interventions that induce AHN in rodents, such as exercise and energy restriction, have been shown to improve the performance of humans in cognitive tasks involving AHN.^141^, ^142^ Yet, many questions regarding the potential mechanisms underlying the relationship between AHN impairment and AD remain unanswered. As outlined in section 8, several micro‐ and macroenvironmental regulators of AHN can be negatively influenced by AD pathology, either through direct or indirect mechanisms. These include mitochondrial function, calcium homeostasis, transcription factors, reactive oxygen species, neurotransmitters, neuronal signaling, and neuroinflammation. Moreover, how the morphology and migration of newborn DGCs in the AHN trajectory might be affected by AD remains unclear. Future research may strengthen the case for the existence of AHN throughout the human lifespan and its impairment in AD. For example, comparing morphology‐based quantifications of proposed human adult immature DGCs between healthy aged and AD patients could provide another layer of evidence for AHN in aged humans and its impairment in AD. In addition, establishing a more detailed transcriptional profile of adult human immature DGCs with a focus on markers of electrophysiological properties such as ion transporters and senescence, could tell us more about the functional state of immature DGCs in health and disease.

As highlighted above, there is strong evidence to suggest that AHN plays a role in AD‐induced cognitive decline. Therefore, strategies to restore or stimulate endogenous AHN could provide effective therapeutic strategies to prevent or slow the progression of the disease. However, given the decline in AHN levels with age, it is unclear whether activation of the remaining stem cells would add sufficient new neurons into the AD brain to prevent cognitive decline. Moreover, AHN will only generate DCGs in the hippocampus and therefore not address the global loss of neurons that occurs in advanced stages of AD. The decline in AHN that occurs during aging might also serve to protect the brain. In particular, the extensive vascularization and mitotic capability in the DG may put this region at elevated risk of tumorigenesis compared to other (post‐mitotic) brain regions.^143^ This is postulated as a reason for differences in levels of aging‐induced AHN between rodents and humans. As our relatively long life span increases the risk of tumorigenesis in the hippocampus, a decrease in AHN during aging might mitigate this risk.^144^ Simply put, impaired performance in tasks such as pattern separation might be the price we must pay to protect our aging brain from the risks of cancer in the hippocampus. This must be considered during the development of any therapeutic strategies aiming to ameliorate hippocampus‐associated cognitive deficits by promoting AHN.

Taken together, contemporary methods currently do not allow for the establishment of causative links between AHN impairment and AD in humans. Yet, they still offer exciting future research directions that will enable us to gain a better mechanistic understanding of AD pathology in the context of AHN.

## CONFLICT OF INTEREST STATEMENT

The authors declare no conflicts of interest. Author disclosures are available in the Supporting information.

## CONSENT STATEMENT

No consent was required for this study.

## Supporting information

Supporting Information
